# Educational Attainment Moderates the Association Between Hippocampal Volumes and Memory Performances in Healthy Older Adults

**DOI:** 10.3389/fnagi.2018.00361

**Published:** 2018-11-08

**Authors:** Deirdre M. O’Shea, Kailey Langer, Adam J. Woods, Eric C. Porges, John B. Williamson, Andrew O’Shea, Ronald A. Cohen

**Affiliations:** ^1^Center for Cognitive Aging and Memory, McKnight Brain Institute, University of Florida, Gainesville, FL, United States; ^2^Department of Clinical and Health Psychology, University of Florida, Gainesville, FL, United States; ^3^Department of Neuroscience, University of Florida, Gainesville, FL, United States; ^4^Brain Rehabilitation Research Center – Malcom Randall Veterans Affairs Medical Center, Gainesville, FL, United States; ^5^Department of Psychiatry, University of Florida, Gainesville, FL, United States

**Keywords:** Hippocampus, episodic memory, cognitive reserve, educational attainment, cognitive aging

## Abstract

**Objective:** To examine whether educational attainment, as a proxy of cognitive reserve, moderated the association between hippocampal volumes and episodic verbal memory performances in healthy older adults.

**Methods:** Data from 76 community dwelling older adults were included in the present study. Measures of hippocampal volumes (total, left, and right) were obtained using FreeSurfer software. Immediate and delayed verbal recall scores were derived from performances on the California Verbal Learning Test-Second Edition and the Wechsler Memory Scale- Third Edition. Educational attainment was defined by years of education. Linear regression analyses were performed using immediate and delayed recall as dependent variables and hippocampal volumes, years of education, and their interaction terms as independent variables. All analyses were controlled for age, sex, depression, and health status.

**Results:** Total and left Hippocampal volumes had a positive main effect on delayed recall only. Additionally, the interaction between total, left, and right hippocampal volumes and education was a significant predictor for delayed recall performance but not for immediate recall performance. The positive association between hippocampal volumes and delayed recall was greatest in those with more years of education.

**Conclusion:** Larger hippocampal volumes were associated with better delayed verbal recall and the effect on delayed recall was greatest in those with more years of education. Having higher levels of education, or cognitive reserve, may enable individuals to capitalize on greater structural integrity in the hippocampus to support delayed recall in old age. However, longitudinal research is needed to investigate the directionality of these associations.

## Introduction

The relationship between advanced age and cognitive decline has been well documented since the beginnings of mental testing in adults (Foster and Taylor, 1920). Episodic memory ability is particularly susceptible to normal aging (Craik and McDowd, 1987; Souchay et al., 2007; Luo and Craik, 2008; Tromp et al., 2015), and is often disproportionately implicated in the early stages of Alzheimer’s disease (AD) (Greene et al., 1996; Fox et al., 1998; Small et al., 2000; Bäckman et al., 2001, 2004; Dubois et al., 2007; Grober et al., 2008; Tromp et al., 2015). Although decades of research has shown that episodic memory is a complex ability involving multiple neural structures, the hippocampus is one of the most well established brain structures involved in episodic memory function (Scoville and Milner, 1957; Markowitsch, 1995; Vargha-Khadem et al., 1997; Desgranges et al., 1998; Squire and Zola, 1998; Tulving and Markowitsch, 1998; Burianova and Grady, 2007; Travis et al., 2014; Gorbach et al., 2017). AD exemplifies the critical role of the hippocampus in memory functioning; AD pathology is characterized by marked hippocampal atrophy in the early stages which correlates to concomitant episodic memory impairment (Bäckman et al., 2001; Kramer et al., 2004; Dubois et al., 2007; Hirni et al., 2013; Tromp et al., 2015). Regarding normal cognitive aging, some cross sectional and longitudinal studies have shown positive associations between episodic memory performance and hippocampal volume (Walhovd et al., 2004; Murphy et al., 2010). These findings would seem to suggest that larger hippocampal volumes are better for memory function. However, contrary evidence that smaller hippocampal volumes are positively associated with episodic memory has been found in healthy young adults (Chantome et al., 1999; Foster et al., 1999). Such evidence points to a dynamic relationship between hippocampal volumes and memory across the lifespan which is consistent with other research (Raz et al., 1998). The mixed findings across the lifespan regarding the hippocampus and memory highlight the point that the relationship between hippocampal volume and memory in older adults is not clear cut (for review see; Van Petten, 2004). Of note, Van Petten (2004) reported that in older samples there was much greater variability in hippocampal-episodic memory associations than in younger samples. While the author suggested that methodological factors such as impact of statistical methods and individual differences in participant characteristics across samples may account for this variability, there may be other moderating factors.

In particular, “reserve” theories (brain and cognitive) have been pivotal in explaining the often discrepant relationship between brain structure and function in the field of aging and dementia. Brain reserve, or passive threshold theories, propose that brain properties (volume, neuronal count, and gray matter density) are better indicators of performance than the presence of pathology (e.g., white matter damage, beta amyloid) (Satz, 1993; Stern, 2012). Brain reserve presupposes cognitive function is altered once the amount of pathology exceeds a threshold that compromises available brain structure. The “active” model of cognitive reserve, a complementary but theoretically distinct concept, proposes that individual differences in functional neural networks allow some people to cope better with pathology than others (Stern, 2012). Lifestyle factors such as greater educational attainment, complex occupations, and engagement in leisure activities have been shown to confer cognitive reserve (Stern, 2002, 2013; Scarmeas and Stern, 2003; Sattler et al., 2012). However, little is still known regarding the underlying neural mechanisms of cognitive reserve and how the expression of reserve varies as a function of age, as well as type of pathology (Barulli and Stern, 2013; Stern et al., 2018). One study directly examined the relationship between cognitive reserve and hippocampal volumes in middle-aged adults. The authors used the total score of the Armed Forces Qualification test to measure cognitive reserve, administered at age 20. This is a 100-item multiple choice paper and pencil test administered to individuals before military induction (Bayroff and Anderson, 1963), and is highly correlated with the Wechsler Adult Intelligence Scale (McGrevy et al., 1974). The authors found that smaller hippocampal volumes were associated with poorer midlife episodic memory, only in those with low cognitive reserve (Vuoksimaa et al., 2013). This is consistent with a compensatory view of cognitive reserve which suggests that higher cognitive reserve may attenuate, the impact of smaller hippocampal volumes on memory function, thus a direct association between hippocampal volume and memory would be expected to be stronger in those with lower cognitive reserve. A more recent study examined whether education modified brain atrophy, as measured by global gray matter volume, on cognitive decline among cognitively normal older adults and individuals with mild cognitive impairment (MCI) and dementia (Mungas et al., 2018). The authors found that higher education accelerated cognitive decline in individuals with greater gray matter atrophy. However, the rate of decline was slower in those with less atrophy but more years of education than individuals with fewer years of education. These results are also consistent with findings from a study which showed a faster rate of cognitive decline in more highly educated individuals with dementia (Stern et al., 1999; Amieva et al., 2014). However, the authors did not examine the impact of education on regional volumes.

To our knowledge, there are no published studies that have directly investigated the role of education/cognitive reserve in the association between hippocampal volumes and verbal episodic memory in cognitively normal older adults.

In the present study we had two primary aims. Firstly, we examined whether hippocampal volumes were associated with episodic verbal memory performances in healthy older adults. Although research has been mixed, findings in older adults have consistently shown greater support for larger hippocampal volumes relating to better episodic memory performances. We therefore hypothesized that larger hippocampal volumes would be positively associated with performances on episodic memory measures. Secondly, we examined whether the association between hippocampal volumes and memory varied as a function of cognitive reserve, as indicated by educational attainment. In line with the findings by Vuoksimaa et al. (2013), we predicted that hippocampal volumes and memory performance would be more strongly and negatively related in individuals with lower levels of cognitive reserve.

## Materials and Methods

### Participants

76 participants (mean age = 71.64, range: 60–93, and 58.8% female) took part in the present study. 92% of the sample were Caucasian (African American = 4, Hispanic = 1, and Other = 1). Participants were recruited from the north-central Florida region via fliers and newspaper advertisements. Data from the present study was selected from a larger study (see; Nissim et al., 2016; O’Shea et al., 2016); participants with full data on variables of interest in the present study were included. Participants provided written and verbal informed consent to take part in the study. An extensive medical history questionnaire was administered to each participant to rule out any age-related or psychiatric disorders. A full neuropsychological test battery was administered to all participants. Cognitive test performances were reviewed by a clinical neuropsychologist to assess for MCI; no participants recruited had performances indicative of MCI. Specific exclusion criteria included: self-reported history of any psychiatric, neurological, or neurodegenerative disorders (e.g., Multiple sclerosis, Alzheimer’s, or Parkinson’s disease). Additional exclusion criteria included whether the participants had any MRI (3T) contraindications (e.g., medical implants or devices, metal in the body, pregnancy or claustrophobia). All study procedures were approved by the University of Florida Institutional Review Board. Characteristics for the study sample are displayed in Table 1.

**Table 1:** Characteristics of the study sample (*n* = 72).

	Mean	SD
Age (years)	71.64	9.72
Sex (% female)	58.8%	–
Education (12–20 years)	16.55	2.55
Physical health sum	1.04	1.00
BDI-II Total Score	3.94	3.67
Hippocampal Volumes	–	–
Total	7,742.45	1,723.94
Right (mm)	3,934.48	861.88
Left (mm)	3,807.97	879.202
CVLT trials 1–4	25.92	5.08
CVLT long delay trial	6.31	2.08
Logical Memory immediate recall	41.57	10.89
Logical Memory delayed recall	24.71	8.15

*CVLT = California Verbal Learning Test; BDI-II: Beck Depression Inventory, 2^nd^ Edition.*

### Neuroimaging Procedure

Magnetic resonance imaging was performed using a 3.0 Tesla (3T) scanner (Achieva; Philips Electronics, Amsterdam, Netherlands) located at the McKnight Brain Institute, University of Florida (Gainesville, FL, United States). The scanner is equipped with a 32-channel receive-only head coil. A high-resolution 3D T1 weighted MPRAGE scan was performed. Measures of hippocampal, entorhinal cortex, and intracranial volumes (ICVs) were obtained with the following scanning parameters: voxel size = 1 mm isotropic; 1 mm slice thickness; TE = 3.2 ms; TR = 7.0 ms; FOV = 240 × 240; number of slices = 170; acquired in a sagittal orientation.

All MRI T1-weighted scans were processed using FreeSurfer software (version 5.3^1^). Regions of Interest (ROI’s; total, left, and right hippocampus volumes) were obtained from the automatic subcortical segmentation stream. Previous research has shown that this method produces reliable and accurate results of subcortical regions (Jovicich et al., 2009; Fischl, 2012). Each scan slice was manually inspected and edited/reprocessed using FreeSurfer (if necessary) by a trained technician. Total, right, and left hippocampal volumes were normalized relative to the individual’s total ICV by dividing each by ICV and multiplying the product by the sample ICV mean.

### Measures

#### Immediate Verbal Recall

*Immediate verbal recall* measures included (i) total correct words recalled over the four trials of the California Verbal learning Test–Second Edition (short form) (CVLT-II) (Delis et al., 1994; Fine et al., 2012), and (ii) Logical memory total immediate recall trial from Wechsler Memory Scale-Third Edition (WMS-II) (Wechsler, 1997). The Immediate memory composite was formed by first converting performances on these two measures to z-scores and summing them.

#### Delayed Verbal Recall

*Delayed verbal recall* performances were assessed using (i) the correct number of words recalled on the long delay free recall trial of the CVLT-II and (ii) the Logical Memory delayed recall trial of the WMS-III. The delayed recall composite was formed by summing the z-scores from these measures.

#### Cognitive Reserve

*Cognitive reserve* was defined as self-reported years of education. Several studies have used years of education as a measure of cognitive reserve (Stern, 2006; Meng and D’Arcy, 2012; Amieva et al., 2014; Harrison et al., 2015). Years of education ranged from 12 to 20 years.

#### Covariates

Previous research has shown that age, sex, medical co-morbidity, and depressive symptoms are associated with decreased hippocampal volumes (Murphy et al., 1996; Debette et al., 2011; O’Shea et al., 2018). Thus, we controlled for these factors in all our analyses. Medical co-morbidity was derived from summing the following variables: smoke cigarettes (yes/no), body mass index >30 (yes/no), arthritis (yes/no), diabetes (yes/no), hypertension (yes/no), and heart failure (yes/no). Depressive symptoms were measured using the Beck Depression Inventory-Second Edition (BDI II; Beck et al., 1996). This 21-item questionnaire is widely used to assess the presence of depressive symptoms over the prior 2 weeks. Each item has a score between 0 and 3. Total scores can range from 0 to 63. The range in the present sample was between 0 and 13 which indicates minimal levels of depressive symptoms.

### Statistical Analyses

All analyses were performed using SPSS version 22 (IBM Corp., Armonk, NY, United States). Figures were derived from the macro “MODPROBE” for SPSS (Hayes and Matthes, 2009). The macro generates all the regression output in addition to estimates of the effects of the primary predictor at the values of the moderating variable for visualizing the interaction term. Moderator values are selected based on the sample mean and plus/minus one standard deviation from the mean. As all variables were converted to z-scores, the mean was zero. Descriptive statistics (mean and standard deviation) of the sample characteristics are presented in Table 1. Correlations between variables are depicted in Table 2. Immediate and delayed recall scores were screened for outliers. Four participants with an immediate recall z-score >3 were removed from analyses. Follow-up analyses run with the inclusion of the participants revealed significant associations that were not present without these participants, thus, these participants were excluded from further analyses. Multiple linear regression analyses were performed to examine the main and interaction effects of education and estimated ICV normalized hippocampal volumes (i.e., total, left, and right) on the immediate and delayed recall memory composites. Age (continuous), sex, BDI-II scores, and the health status summed score were used as covariates in all of the models. A total of six regression analyses were performed. False Discovery Rate (FDR) adjustment for multiple comparisons was conducted within the R-statistics package for the set of six regression analyses using the *p.adjust* function. This FDR correction is reflected in the in-text results presented below and noted in Table 3. All variables were z-scored to facilitate the interpretation of effects. Alpha was set at ≤0.05.

**Table 2:** Results from regression analyses.

Outcome	Immediate recall	Delayed recall
	B (*p*-value)	B (*p*-value)
Total hippocampal volume	**0.699 (*p* = 0.031,** FDRp = 0.053**)**	**0.294 (*p* = 0.020, FDRp = 0.048)**
Interaction with education	0.392 (*p* = 0.133, FDRp = 0.143)	**0.124 (*p* = 0.008, FDRp = 0.039)**
Left hippocampal volume	**0.463 (*p* = 0.029,** FDRp = 0.053**)**	**0.309 (*p* = 0.013, FDRp = 0.039)**
Interaction with education	0.241 (*p* = 0.139, FDRp = 0.143)	**0.232 (*p* = 0.013, FDRp = 0.039)**
Right hippocampal volume	**0.446 (*p* = 0.043,** FDRp = 0.057**)**	**0.260 (*p* = 0.042,** FDRp = 0.057**)**
Interaction with education	0.248 (*p* = 0.143, FDRp = 0.143)	**0.254 (*p* = 0.007, FDRp = 0.039)**

*All analyses were controlled for age, gender, depression, and health status; Significant associations shown in bold; FDRp = False discovery adjusted p-value.*

**Table 3:** Pearson’s Correlations between independent variables.

	Age	education	sex	Physical health	BDI-II	T_hipp	L_hipp	R_hipp
age	1							
Education	-0.025	1						
Sex	0.085	-0.165	1					
Physical health	0.183	0.038	-0.095	1				
BDI-II	0.327^∗∗^	-0.253 ^∗^	-0.186	0.137	1			
T_hipp	-0.462^∗∗∗^	-0.298^∗∗^	0.362^∗∗∗^	-0.150	-0.315^∗∗^	1		
L_hipp	-0.435^∗∗∗^	-0.312^∗∗^	0.376^∗∗∗^	-0.137	-0.306^∗∗^	0.991^∗∗∗^	1	
R_hipp	-0.462^∗∗∗^	-0.298^∗∗^	0.342^∗∗^	-0.159	-0.318^∗∗^	0.991^∗∗∗^	0.963^∗∗∗^	1

## Results

### Immediate Recall

After FDR correction, the main effects of total, right, and left hippocampal volumes were no longer significant. The education by hippocampal volume interaction terms were also not statistically significant in any of the models.

### Delayed Recall

#### Total Hippocampal Volume

**FIGURE 1:**
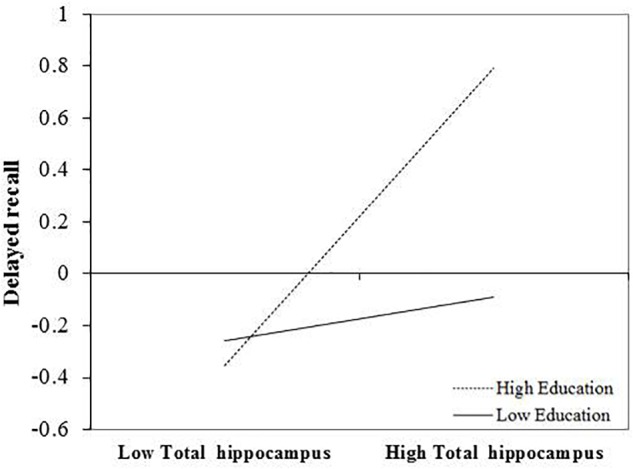
Effect of total hippocampal volume on delayed recall at the values of the moderator education.

After FDR correction, the positive and significant main effect of total hippocampal volume on delayed recall performance remained significant *b* = 0.294, *t*(72) = 2.38, and *p* = 0.048. The interaction between education and total hippocampal volume was also statistically significant following correction, *b* = 0.124, *t*(72) = 2.72, and *p* = 0.039. The inclusion of the interaction term explained an additional 8% of the total model variance, *R*^2^ = 0.31, *F*(7,65) = 4.154, and *p* < 0.001. The interaction revealed that larger total hippocampal volumes were associated with better delayed memory performances and that this effect was greatest in those with higher levels of education (Figure 1).

#### Left Hippocampal Volume

**FIGURE 2:**
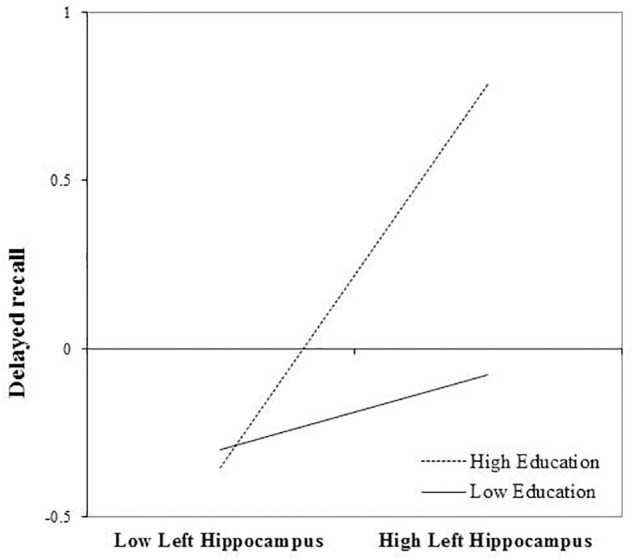
Effect of left hippocampal volume on delayed recall at the values of the moderator education.

After FDR correction, the main effect of left hippocampal volume was a significant predictor of delayed recall performance, *b* = 0.309, *t*(72) = 2.56, and *p* = 0.039. The interaction between education and left hippocampal volume was also statistically significant, *b* = 0.232, *t*(72) = 2.56, and *p* = 0.039. The positive association between left hippocampal volumes and delayed recall was greatest in those with more years of education (Figure 2). The inclusion of the interaction term accounted for an additional 7% of the total model variance, *R*^2^= 0.31, *F*(7,65) = 4.15, and *p* < 0.001.

#### Right Hippocampal Volume

**FIGURE 3:**
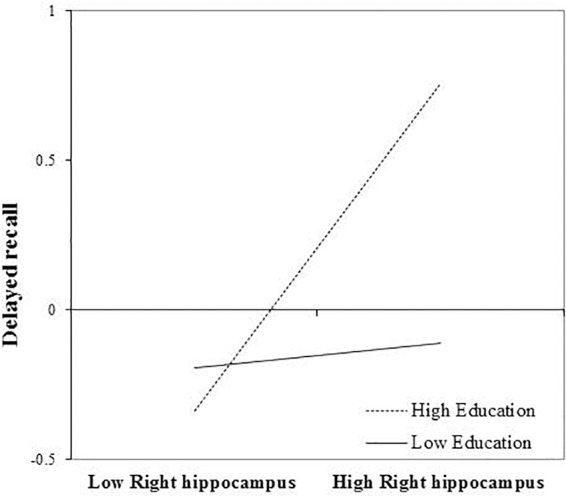
Effect of right hippocampal volume on delayed recall at the values of the moderator education.

After FDR correction, the main effect of right hippocampal volume on delayed recall was not statistically significant, *b* = 0.260, *t*(72) = 2.080, and *p* = 0.057. The interaction between education and right hippocampal volume remained significant, even after FDR correction, *b* = 0.254, *t*(72) = 2.80, and *p* = 0.039. Again, revealing that the positive association between right hippocampal volume and delayed recall was greatest in those with more years of education (Figure 3). The inclusion of the interaction term explained an additional 8% of the total variance in the model, *R*^2^ = 0.30, *F*(7,65) = 7.84, and *p* < 0.01.

## Discussion

The results from the present study revealed that greater total, right, and left hippocampal volumes were associated with better performances on delayed recall measures. A trend for significance was revealed between greater hippocampal volumes and better immediate memory performances, following FDR correction. Additionally, we found that higher educational attainment in conjunction with greater hippocampal volumes (total, left, and right) was associated with better delayed but not immediate recall performances. In sum, the positive main effect between larger hippocampal volumes and memory performances was strongest in those with more years of education.

These findings are contrary to initial predictions: that similar to the findings of Vuoksimaa et al. (2013) in middle-aged adults, the association between smaller hippocampi and memory performances would be stronger for those with lower cognitive reserve/years of education. Previous studies have also shown that higher cognitive reserve attenuates the direct association between brain structure and function in clinical samples (Solé-Padullés et al., 2009; Bosch et al., 2010; Franzmeier et al., 2017a), so we expected that the association between smaller hippocampal volumes and memory would be stronger in those with lower education. One possibility that may account for differing findings is that the mean level of education in Vuoksimaa et al’s. (2013) study was much lower (12.91 years) compared to the present study sample (16.55 years, range 12–20 years). It may be that if individuals with much lower levels of education were included in the study, a stronger effect at this level may have been evident. The restricted representation of varying levels of cognitive reserve is a limitation to interpreting these findings, as the two studies cannot be directly compared. However, investigating the impact of varying levels of cognitive reserve amongst highly educated older individuals has merit. Indeed, previous studies have shown that more highly educated older adults (i.e., >12 years) with AD pathology follow a much steeper cognitive decline once they meet their disease burden threshold (Scarmeas et al., 2006; Meng and D’Arcy, 2012; Stern, 2012, 2013) and that higher education accelerates cognitive decline in individuals with greater brain atrophy (Mungas et al., 2018). Furthermore, early detection of cognitive decline may be most challenging in a highly educated population which may limit opportunities for early intervention. Prior research has also shown that declines in fluid, or age-sensitive cognitive abilities, including episodic memory may be masked in individuals with more cognitive reserve (Stern, 2009; Wilson et al., 2009; Giogkaraki et al., 2013).

Further consideration of the cognitive reserve hypothesis is provided to explain the discrepant findings between the present study and Vuoksimaa et al’s. (2013) study. Using proxies of cognitive reserve as moderating variables in the analyses of the relationship between brain volume and cognition seems straightforward; however, the mechanisms underlying cognitive reserve as it relates to (i) varying levels and types of pathology and (ii) brain reserve are not clearly understood (Stern et al., 2018). The uncertainty regarding such mechanisms may complicate the interpretation of findings in the context of these variables. Stern et al. (1994); Stern (2009) hypothesizes that there are two facets of cognitive reserve: (i) neural reserve which relates to inherent individual differences in neural network capacity to perform cognitively demanding tasks, and (ii) neural compensation wherein the effects of brain pathology and/or aging alter brain structure in a way that necessitates the recruitment of alternative networks to perform at the same level of cognitive functioning. Although brain and cognitive reserve are theoretically distinct concepts, they may also be considered as complementary or interactive. A review of this topic by Arenaza-Urquijo et al. (2015) offers an integrative perspective on reserve theories that emphasizes the interactive relationship between brain reserve and cognitive reserve. Firstly, compensatory neural networks (cognitive reserve) cannot exist without being substantiated by an anatomical substrate (brain reserve). Thus, the degree of cognitive reserve is likely altered by the integrity of that anatomical substrate. In other words, more brain reserve may serve to optimize cognitive reserve. This effect may be especially apparent in more highly educated, cognitively normal older adults as represented by the present study sample. Secondly, proxies of cognitive reserve such as education, leisure activities, and occupation have been shown to alter both brain structure and functional connectivity across studies (Whalley et al., 2004; Bartres-Faz et al., 2009; Solé-Padullés et al., 2009; Franzmeier et al., 2017a; Tang et al., 2017).

In animal models of normal aging, environmental enrichment, theorized to confer cognitive reserve, has been shown to: promote neurogenesis and synaptic strength in the hippocampus, potentiate synaptic plasticity, and enhance brain derived neurotrophic factor (BDNF) expression (Gelfo et al., 2018). Cognitive reserve, quantified by “residualized memory” (i.e., the variance in cognition that is not explained by demographics and brain predictors of cognition (Stern et al., 2018), has additionally been found to be associated with left frontal cortex (LFC)-connectivity during successful episodic memory performance in healthy older adults (Franzmeier et al., 2017b). In persons with MCI, higher residual memory and education were additionally related to greater resting-state connectivity of the LFC to the default mode network and default attention network (Franzmeier et al., 2017c). Together, these findings further support the dynamic relationship between measures of cognitive reserve and brain structure/function in aging. However, little is still known how brain structure in healthy aging alters the association between proxies of cognitive reserve and cognition in normal aging. Specifically, the association between hippocampal volumes and education in older age is not clearly understood. In one study, education was directly associated with larger hippocampal volumes (Noble et al., 2012), although this effect was not significant in adjusted models. However, other studies have not shown an association between hippocampal volumes and education (Noble et al., 2012; Staff et al., 2012). Education was negatively correlated with hippocampal volumes in the present study; an association that was no longer significant in the adjusted models, however. The association between education and hippocampal volumes is further complicated by evidence showing the changing role of education on brain volume; that is, higher education was shown to slow rate of cognitive decline when atrophy was minimal (in normal older adults) but accelerates cognitive decline when atrophy is more extensive (i.e., in dementia) (Mungas et al., 2018). Drawing on the work of Arenaza-Urquijo et al. (2015), these findings are in line with the proposal that the role or expression of cognitive reserve may change as a function of increasing pathology and presumably, age-related brain changes. Neural reserve or innate individual differences in neural processing, as described by Stern (2009), may be the predominant determinant for individual differences in cognitive function in healthy older adults. Neural compensation, which may emerge only in the context of pathology or in much later life, and therefore, may override innate differences and result in an alternative expression of cognitive function. It may be that in healthy older adults higher levels of cognitive reserve allow an individual to capitalize on greater brain structure. In sum, evidence suggests that the effect of education on cognition changes as a function of degree of brain pathology/health. From this perspective, our findings are in line with what would be expected in a healthy sample, where we would expect the positive effect of education on memory performances to be greatest in individuals with minimal levels of hippocampal volumes loss. However, there are several key difference between the present study and Mungas et al’s. (2018) study including the cross sectional design (i.e., we did not measure rate of decline), range of education and measure of brain volume so the present study’s findings are not directly comparable. Future research is needed to explore longitudinal associations between brain volume and rate of cognitive decline among healthy older adults with varying levels of education to further investigate the role of cognitive reserve in cognitively normal older adults.

There are several limitations to the present study that should be noted. Firstly, as referred to earlier, the range of education in the present study sample is limited (i.e., 12–20), resulting in a mean education level (i.e., 16 years) that is relatively high and not representative of the U.S. population. Additionally, the limited range of education does not represent a subgroup of individuals with lower levels of cognitive reserve. Therefore, generalizability of the study results are limited and more specific to highly educated, predominantly Caucasian older adults. However, investigating the relationship between brain structure and function in a more highly educated group is of value given prior evidence that this population may follow a faster cognitive decline once an age-related disease (e.g., AD) burden threshold is reached (Stern et al., 1999; Scarmeas et al., 2006; Meng and D’Arcy, 2012; Stern, 2012). Although our sample consisted of healthy older adults, there is a paucity of research examining this subgroup of the population in the context of how cognitive reserve alters brain and behavior relationships. Future research should include individuals with a wider range of educational attainment to examine and compare different levels of cognitive reserve and how that may moderate the association between hippocampal volume and episodic memory performance. As education is also a proxy of social economic status (Mirowsky, 2017) including individuals with a wider range of education may also influence the pattern of other sample characteristics such as health status or level of depressive symptoms. Poorer health and greater depressive symptoms have been associated with lower SES and/or educational attainment (Mirowsky, 2017). Poor physical health and greater depressive symptoms can negatively impact memory in older adults (Serper et al., 2014; Donovan et al., 2017), thus, further necessitating the inclusion of a more educationally diverse sample to increase generalizability. Additionally, the majority of participants in the present study were Caucasian which also limits the generalizability of findings. A more ethnically diverse sample would be needed to generalize these findings to the U.S. population. The cross sectional design is an obvious limitation in so far as understanding whether greater educational attainment increases hippocampal volumes or whether greater brain volume increases the likelihood of greater educational attainment. There may be a bi-directional or synergistic effect between structural integrity and activity engagement/lifetime exposures (for reviews see; Whalley et al., 2004; Nyberg et al., 2012; Barulli and Stern, 2013). However, establishing the directionality of the association between education and hippocampal volumes was not the focus of the present study. Instead we were interested in showing whether the association between this region varied as a function of education level. Future research incorporating an explanation of how these two variables are related would advance this topic further.

The findings from the present study show that larger hippocampal volumes are related to better delayed verbal recall performance. Additionally, those with more years of education and larger hippocampal volumes had better delayed recall performances. The moderating role of education on the association between hippocampal volume and memory has not been well researched in older adults. Our findings further revealed that even in a more highly educated sample, on average there is still an effect of varying levels of education. The potential clinical implication of the present findings is that increasing cognitive reserve may optimize structural integrity and promote optimal cognitive performance in healthy older adults. However, longitudinal studies are necessary to understand the directionality of these associations. Additionally, investigating whether other non-static proxies of cognitive reserve, e.g., leisure activities, have a similar effect and whether higher cognitive reserve modifies rate memory decline in cognitive normal older adults is needed.

## Author Contributions

DO was involved in all aspects of the manuscript including hypothesis formation, statistical analyses, and writing of the manuscript. AO conducted volumetric analyses that were used in the manuscript. KL, EP, JW, AW, and RC were involved in the conception of the study. RC was involved in the conception of the manuscript. All authors contributed to the intellectual content and approved the final version to be published.

## Conflict of Interest Statement

The authors declare that the research was conducted in the absence of any commercial or financial relationships that could be construed as a potential conflict of interest.

## Footnotes

1 http://surfer.nmr.mgh.harvard.edu
